# Alkaloid from *Alstonia yunnanensis* diels root against gastrointestinal cancer: Acetoxytabernosine inhibits apoptosis in hepatocellular carcinoma cells

**DOI:** 10.3389/fphar.2022.1085309

**Published:** 2023-01-11

**Authors:** Qi Lai, Chun-Ju Yang, Qi zhang, Min Zhuang, Yan-Hua Ma, Cheng-Yuan Lin, Guang-Zhi Zeng, Jun-Lin Yin

**Affiliations:** ^1^ Key Laboratory of Chemistry in Ethnic Medicinal Resources, State Ethnic Affairs Commission & Ministry of Education, School of Ethnic Medicine, Yunnan Minzu University, Kunming, China; ^2^ Centre for Chinese Herbal Medicine Drug Development Limited, Hong Kong Baptist University, Hong Kong, China; ^3^ School of Chinese Medicine, Hong Kong Baptist University, Hong Kong, China

**Keywords:** hepatocellular carcinoma cells, *Alstonia yunnanensis* Diels, indole alkaloid, proliferation, apoptosis

## Abstract

Liver cancer belongs to Gastrointestinal (GI) malignancies which is a common clinical disease, a thorny public health problem, and one of the major diseases that endanger human health. Molecules from natural products (NPs) or their derivatives play an increasingly important role in various chronic diseases such as GI cancers. The chemical composition of the *Alstonia yunnanensis Diels* roots was studied using silica column chromatography, gel chromatography, recrystallization, and HPLC, and the compounds were structurally identified by modern spectral analysis using mass spectrometry (MS) and nuclear magnetic resonance (^1^H-, ^13^C-, HMQC-, HMBC-, and ^1^H-^1^HCOSY-NMR), ultraviolet and visible spectrum (UV), and electronic Circular Dichroism (ECD). Acetoxytabernosine (AC), an indole alkaloid with antitumor activity, was isolated from *Alstonia yunnanensis Diels* root. The current study aimed to investigate the influence of AC on the cell proliferation of BEL-7402 and SMMC7721 and to elucidate the underlying mechanism. The absolute configuration of AC was calculated by ECD (electronic circular dichroism). The effects of AC on the viability of different tumor cell lines were studied by the SRB method. The death mode of human hepatoma cells caused by AC was studied by TUNEL cell apoptosis detection and AnnexinV-FITC/PI double staining image. Mitochondrial membrane potential was detected by JC-1. The effects of AC on the expression of apoptosis-related proteins (Caspase9, Caspase3, and Parp-1) in SMMC7721 and BEL-7402 cells were detected by western blot. It was found that the absolute configuration of AC is 19(s), 20(s)-Acetoxytabernosine. AC could induce apoptosis of SMMC7721 and BEL-7402, and block the replication of DNA in the G1 phase. Under the treatment of AC, the total protein expression of apoptosis-related proteins (Caspase9, Caspase3, and Parp-1) significantly decreased in SMMC7721 and BEL-7402. The results suggested that AC induced apoptosis through a caspase-dependent intrinsic pathway in SMMC7721 and BEL-7402, and natural product-based drug development is an important direction in antitumor drug discovery and research.

## Introduction

Gastrointestinal (GI) tumors are common malignancies, and the high incidence, recurrence, and metastasis rates, limited drug choices and poor treatment outcomes have caused problems for clinicians and patients (Parab, DeRogatis et al., 2019; Zhu, Zhu et al., 2021). Currently, chemotherapy and targeted therapies are the mainstays of GI tumors, and the advent of immunotherapy has also given patients with GI tumors a ray of hope (Sun and Karin, 2014; Lee, Tan et al., 2018; Joshi and Badgwell, 2021). Liver cancer is one of the GI tumors, mainly because the liver is also involved in the functioning of the body’s digestive system, in which it continuously promotes the production of bile to aid digestion (Siegel, Miller et al., 2020). Liver cancer is the sixth most prevalent cause of cancer and the fourth most common cause of cancer-related mortality globally, and liver cancer is a common clinical disease, a thorny public health problem, and one of the major diseases that endanger human health (Bray, Ferlay et al., 2018; Yang, Hainaut et al., 2019). At present, the main treatment methods for liver cancer and tumors are surgery, chemotherapy, and other treatments (Zhao, Gray et al., 2019). However, in the limited drug selection, drug resistance is often the reason for the treatment failure of tumor patients, and the mechanism is a complex process (Li and Raut, 2019). Therefore, there is an urgent need to develop more cytotoxic drugs for cancer.

Liver cancer development and progression are regulated by multiple genes and pathways, with uncontrolled proliferation and apoptotic inactivation (Farazi and DePinho, 2006; Whittaker, Marais et al., 2010; Dapito, Mencin et al., 2012). Thus, inhibiting the growth of liver cancer cells and inducing apoptosis is one of the main goals of liver cancer treatment. The regulatory process of apoptosis involves various apoptosis-related pathways such as Bcl-2, Bax, Caspase, and ROS, and targeting apoptosis is a promising therapeutic strategy in the treatment of liver cancer (Chien, Chang et al., 2005; Jiang, Chen et al., 2017; Wang, Mang et al., 2022). Previous studies have reported that most anti-liver cancer treatments will touch the induction of apoptosis and related cell death networks, and the active ingredients of natural products (NPs) can significantly induce apoptosis of liver cancer cells (Kim, 2016; Liu, Li et al., 2020), and both morusin (Gao, Wang et al., 2017) and amygdalin (Huang, Li et al., 2017) can induce apoptosis in liver cancer cells. Therefore, exploring the anti-tumor mechanism of action of natural product active ingredients is an important way to screen the action targets of tumor therapeutics.

Historically, NPs have played a key role in drug discovery, including cancer and infectious diseases (Atanasov, Waltenberger et al., 2015; Atanasov, Zotchev et al., 2021), cardiovascular disease, and multiple sclerosis. Considerable attention has therefore been given to developing NPs strategies to vanquish tumor growth (Waltenberger, Mocan et al., 2016; Tintore, Vidal-Jordana et al., 2019). NPs are an important resource for exploring new anti-tumor drugs, which are rich in multi types of active ingredients (Newman and Cragg, 2016), such as polysaccharides (Chihara, Hamuro et al., 1970), flavonoids (Lv, Lai et al., 2022), terpenes (Zeng, Wang et al., 2018), and alkaloids (Zhang, Qiang et al., 2019), which could kill or inhibit cancer cells, and are characterized by multi-target and link effects. As for alkaloids, those with antitumor effects include camptothecin (CPT) (Chen, Wang et al., 2017), matrine (Ye, Zou et al., 2018), vincristine (Potier, 1989), harringtonine (Jiang, Liu et al., 1983), and berberine (BR) (Goto, Kariya et al., 2012), with a wide variety of anti-tumor activities and mechanisms. Previous research has confirmed that indole alkaloids such as vinblastine and vincristine were isolated from vinca plants of the Apocynaceae family and widely used in clinical treatment (Johnson, Wright et al., 1960; Bohannon, Miller et al., 1963).

*Alstonia yunnanensis* Diels produced in Yunnan province (China) belongs to the genus *Alstonia* (Feng, Li et al., 2009). Previous studies have found that *Alstonia yunnanensis* Diels is rich in indole alkaloids which with anti-tumor activity and anti-inflammatory (Feng, Li et al., 2009; Li, Chen et al., 2017). Furthermore, in folk, it can also cure a headache and reduce swelling, and even blood pressure. We have previously isolated some indole alkaloids from *Alstonia yunnanensis* Diels, including a new indole alkaloid, and reported the antitumor activity of these compounds (Lai Qi, 2021). In the present study, the indole alkaloid Acetoxytabernosine (AC) was isolated and identified from the *Alstonia yunnanensis* Diels for the first time using silica column chromatography, gel chromatography, recrystallization, and chemical structure determined by ^1^D, ^2^D NMR, MS, UV, and ECD, and there is no report on its activity on hepatocellular carcinoma cells. Our cytotoxicity screening of AC on hepatocellular carcinoma, colon, and gastric cancer cells revealed that AC was effective in inhibiting proliferation and inducing cell cycle arrest in hepatocellular carcinoma cells. By using the SMMC7721 and BEL-7402 cells, we found that the apoptosis inhibitor was effective in reversing the toxicity of AC on hepatocellular carcinoma cells. In brief, this study explores the effect of AC on the proliferation of hepatocellular carcinoma and further explores its anti-tumor mechanism, which provides a reference for the study of the pharmacological activity of *Alstonia yunnanensis* Diels.

## Materials and methods

### Reagents

FBS, RPMI1640 medium, glutamine, and DMEM medium were purchased from Biological Industries (Israel). Ferrostatin-1 (Fer-1), Liproxstatin-1 (Lip-1), Z-VAD-FMK (Zvad), and Necrostatin (Nec-1) were purchased from MedChemexpress (MCE) in the United States. AnnexinV/PI cell double staining apoptosis detection kit, TUNEL (ALEX647) apoptosis detection kit, cell cycle detection kit, and DAPI anti-fluorescence quenching mounting tablets were purchased from Shanghai Yisheng Biotechnology Co. Ltd. Sulfonyl Rhodamine B (SRB) was purchased from Shanghai Aladdin Reagent Co. Ltd. Tris (Tris) was purchased from Shanghai Shenggong Biological Engineering Co. Ltd. Trichloroacetic acid (TCA) was purchased from Shanghai Saen Chemical Technology Co. Ltd. Anti-Caspase3, anti-Caspase9, anti-Parp-1, anti-Cleaved-parp-1 were purchased from Shanghai Shenggong BBILIFE. Anti-Cleaved-caspase9 was purchased from Absin (Shanghai, China). Anti-active-Caspase3 was purchased from ProteinTech (Chicago, United States). All consumables are purchased in NEST Biotechnology (Wuxi, China).

### Materials

In this experiment, *Alstonia yunnanensis* Diels was obtained from Changshan Mountain Yongde County, Lincang City, Yunnan Province. The plant samples (JGCC-2018-01) were collected and identified by Kunming Caizhi Biotechnology Co. Ltd. Plant specimens were preserved in the Key Laboratory of Chemistry in Ethnic Medicinal Resources.

### Equipment

IMARK multifunctional microplate reader (Bio-Rad, United States). 1658001 vertical electrophoresis belt transfer system (Bio-Rad, United States). Tanon 5200 chemiluminescence imager (Tianneng, China). DMIL-LED trinocular inverted microscope (Leica, Germany). TCS-SP8 laser confocal microscope (Leica, Germany). Thermo Scientific 3425 carbon dioxide cell incubator (Thermo Scientific World Er, United States). Thermo TSU-600V -80°C ultra-low temperature refrigerator (Thermo Fisher, United States). Bruker AV nuclear magnetic resonance instrument (Bruker, Germany). Waters AutoSpec Premier P776 high-resolution magnetic mass spectrometer (Waters, United States). The pipette and Eppendr of 5424R centrifuge (Eppendorf, Germany).

### Separation process

2.9 kg of dried *Alstonia yunnanensis* Diels root was extracted with methanol to obtain 150 g of extract. The extract was suspended in an appropriate amount of water, first extracted with an appropriate amount of petroleum ether (PE), then the aqueous phase was adjusted pH to 3-4 with glacial acetic acid, and then extracted with ethyl acetate (EA) to obtain 32 g of part **A**. Next, the aqueous phase of the EA extraction was adjusted to a pH of approximately 10 with ammonia, and the EA was re-extracted to obtain 7.2 g of part **B**. 60-100 mesh silica gels 150 g mixed with part A using column chromatography purification with PE/EA (50:1 to 10:1) to obtain 9 g Fr.3 elution segment. Then, these 9 g samples are separated by Sephadex LH-20 chromatography and detected by TLC. The components were combined with the same polarity to obtain a 30 mg component (Fr.3.2). This component was eluted by 300 mesh silica gel columns according to PE/EA (50:1 to 20:1) as the mobile phase, and TLC detection to combine the same components. Then, this part was recrystallized with methanol as the solvent to obtain 17.2 mg of compound Acetoxytabernosine (AC). Similarly, 60-100 mesh silica gels 50 g mixed with part B using column chromatography purification with PE/EA (30:1 to 10:1) to obtain 513 mg Fr.Ш elution segment. Fr.Ш was separated by PE/EA (50:1 to 5:1) to get 100 mg Fr.Ш.2. Thereafter, Fr.Ш.2 was separated by gel chromatography, and the same polarity was combined by TLC and then eluted by HPLC with MeOH/H_2_O (100:1 to 20:1) to get 15.3 mg AC.

### Structural identification and ECD calculation of compounds

1 and 2-dimensional NMR experiments were recorded on a Bruker DRX-600 spectrometer operating at 400 MHz (^1^H) and 100 MHz (^13^C) at 300 K (chemical shifts δ in ppm, coupling constants J in Hz) (Bruker, Germany). Mass spectra of AC were obtained on a Waters AutoSpec Premier P776 mass spectrometer (Waters Co., Milford, MA, United States). In this experiment, the ECD calculation is done by the research dog instrument testing platform. ECD Calculation The calculations for ECD spectra were conducted as published previously (Shi et al., 2019).

### Anticancer activity assay *in vitro*

Cells BEL-7402, N87, AGS, CT26, HCT116, and SMMC7721 were provided by the National Collection of Authenticated Cell Cultures. After the cells were resuscitated at 37°C, they were cultured in RPMI1640/DMEM containing 10% FBS, in a 37°C, 5% CO2 incubator, and passaged every 2 days. Logarithmic growth phase cells were seeded into a 96-well culture plate (5000 cells/well) and medicine administration was conducted for 48 h. SRB (0.1%) was used to stain the cells, and the wavelength of 515 nm was used to detect the absorption peak.

### TUNEL cell apoptosis detection

SMMC7721 and BEL-7402 (5000 cells) grow on covering glass for 24 h. Then, according to AC 50 μM, and AC 50 μM with Zvad 10 μM co-administered for 24 h. After the cells were fixed with PBS with paraformaldehyde at 4°C for 25 min, aspirate the supernatant, and treat the sample with 2 μg mL^−1^ of proteinase K for 5 min to make the cell permeable. Next, cells were fixed with 100 μL of 1×Equilibration Buffer for 10-30 min, aspirated the supernatant and incubated with Alexa Flour 647-12-dUTP Labeling Mix for 1 h at 37°C, washed away the background with PBS, and sealed the cell slide with DAPI-containing sealing agent. The pictures were observed and taken under a laser confocal microscope.

### Flow cytometry cell apoptosis detection

Flow cytometry was used to detect the total cell apoptosis rate induced by AC on SMMC7721 and BEL-7402, which were treated with AC and apoptosis inhibitors (Zvad) for 24 h, and the results were analyzed by prism 8.0. According to the experimental grouping, the cells were collected directly into 1.5 ml centrifuge tubes, the number of cells per sample was about 2×10^6^, centrifuged for 5 min, and the medium was discarded. The cells were washed once with PBS and centrifuged at 500–1000 r/min for 5 min. The cells were resuspended with 100 μL of the labeling solution and incubated for 10-15 min at room temperature in the dark. Next, the cells were centrifuged at 500–1000 r/min for 5 min and washed the cells with PBS once. Then, the incubation buffer solution was added and incubated for 20 min at 4°C, avoiding light and shaking softly. The excitation light wavelength of the flow cytometer was 488 nm, a band-pass filter with a wavelength of 515 nm was used to detect FITC fluorescence, and a filter with a wavelength greater than 560 nm was used to detect PI.

### Flow cytometry cell cycle detection

The cell cycle was detected by Flow cytometry, and cells were seeded into a 6-well plate. After the AC treatment, the cells were collected and fixed with 70% ethanol at 4°C for more than 2 h, centrifuged at 1500 RPM, and discarded the ethanol fixative. Then, the cells were washed with PBS and centrifuged to discard the PBS. At last, the cells were suspended with the fixative solution, and incubated with RNase and PI at 37°C in the dark for 30 min. Flow cytometry detects cell cycle distribution, and the final data of Flow cytometry were processed by Modfit TL 4.1 software.

### JC-1 testing

Cells were collected and the final spreading density was 2×10^5^ cells/well in 6-well plates with 2 ml per well. The final concentration of AC was 50 μM and the final concentration of colchicine was 1.5 μM. The plates were gently shaken and incubated for 4 h at 37°C in a constant temperature incubator with 5% CO_2_. Cells were collected for JC-1 staining (beyotime, China), incubated for 15 min Centrifuged and the supernatant discarded. Cells were resuspended again and flow cytometry was performed immediately.

### Western blot assay

Western blot was used to detect the expression of proliferation and metastasis-related proteins. SMMC7721 and BEL-7402 were treated with AC at three concentrations of 25, 50, and 100 μM. SMMC7721 and BEL-7402 were treated with AC for 24 h. The cells were digested and collected to lysis with ultrasound for 5 min. The protein concentration was determined by the coomassie brilliant blue method. Polyacrylamide gel electrophoresis was performed with a 50 μg protein mixture in each electrophoresis hole. Then the protein was transferred to the membrane. Subsequently, the membranes were incubated in TBST (Tween-20) containing 5% skim milk at 25°C. The membranes were rinsed with TBST and then incubated overnight with primary antibodies Parp-1, Cleaved-parp-1, Caspase9, Cleaved-caspase9, Caspase3, and Active-caspase3 using β-actin as the internal reference protein. Thereafter, the membranes were rinsed with TBST and incubated with a secondary antibody for 1 h. ECL reaction, darkroom development, exposure. ImageJ software analyzed the ratio of the protein content of each electrophoresis band of the protein content of β-actin.

### Statistical methods

Prism 8.0 was used to perform statistical analysis of the data. The results of each experiment were independently repeated 3 times. Data are shown as mean ± standard error of the mean (SEM). Student’s t-test and One-way ANVOA were used to calculate statistical differences between groups. *p* < 0.05 was considered significant.

## Result

### AC extraction, separation and identification process

**FIGURE 1:**
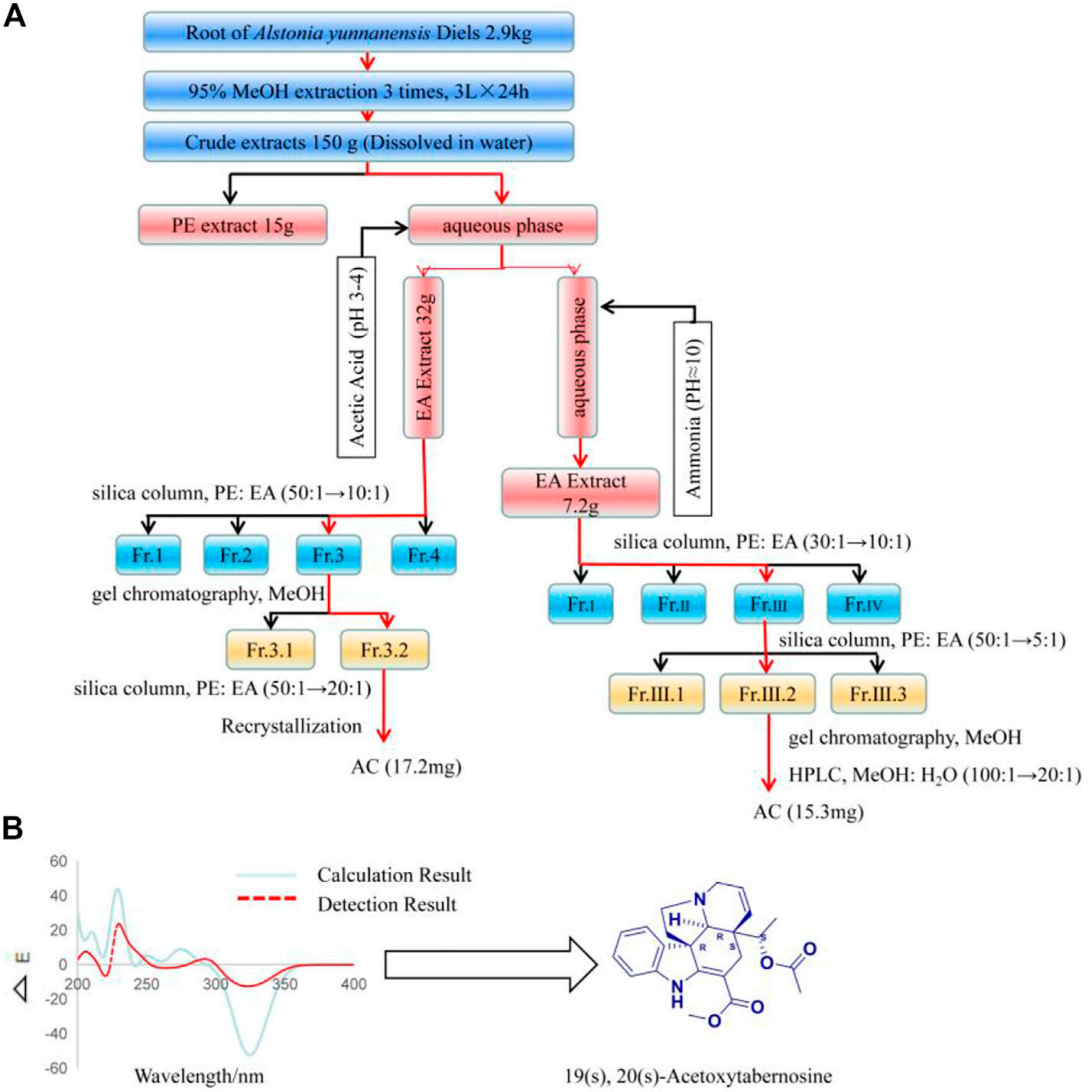
AC isolated from the *Alstonia yunnanensis* Diels root. **(A)** The extraction and isolation process of AC. **(B)** Experimental ECD spectra of AC, and the absolute configuration is 19(s), 20(s) -Acetoxytabernosine.

AC was separated from the *Alstonia yunnanensis* Diels root, and the compound was structurally identified by modern spectral analysis using MS and NMR, and UV (Supplementary Figures S1–S8). As shown in Figure 1A, 17.2 mg AC was isolated from part A and 15.2 mg AC was obtained from part B, involving silica column chromatography, gel chromatography, recrystallization, and HPLC. In addition, modern wave spectroscopy to identify the chemical structure of AC, The ^1^H NMR, and ^13^C-NMR data were consistent with Acetoxytabernosine. The mass spectrometry of AC [M + H]^+^ is 395 (m/z), [M + Na]^+^ is 419 (m/z), and AC was calcded for 394.19. In addition, compared with the detection result, 19(s), 20(s)-Acetoxytabernosine has a positive cotton effect at 270-300. The absolute stereochemistry calculated by ECD is 19(s), 20(s)-Acetoxytabernosine (Figure 1B).

### AC inhibits proliferation of GI tumours

**FIGURE 2:**
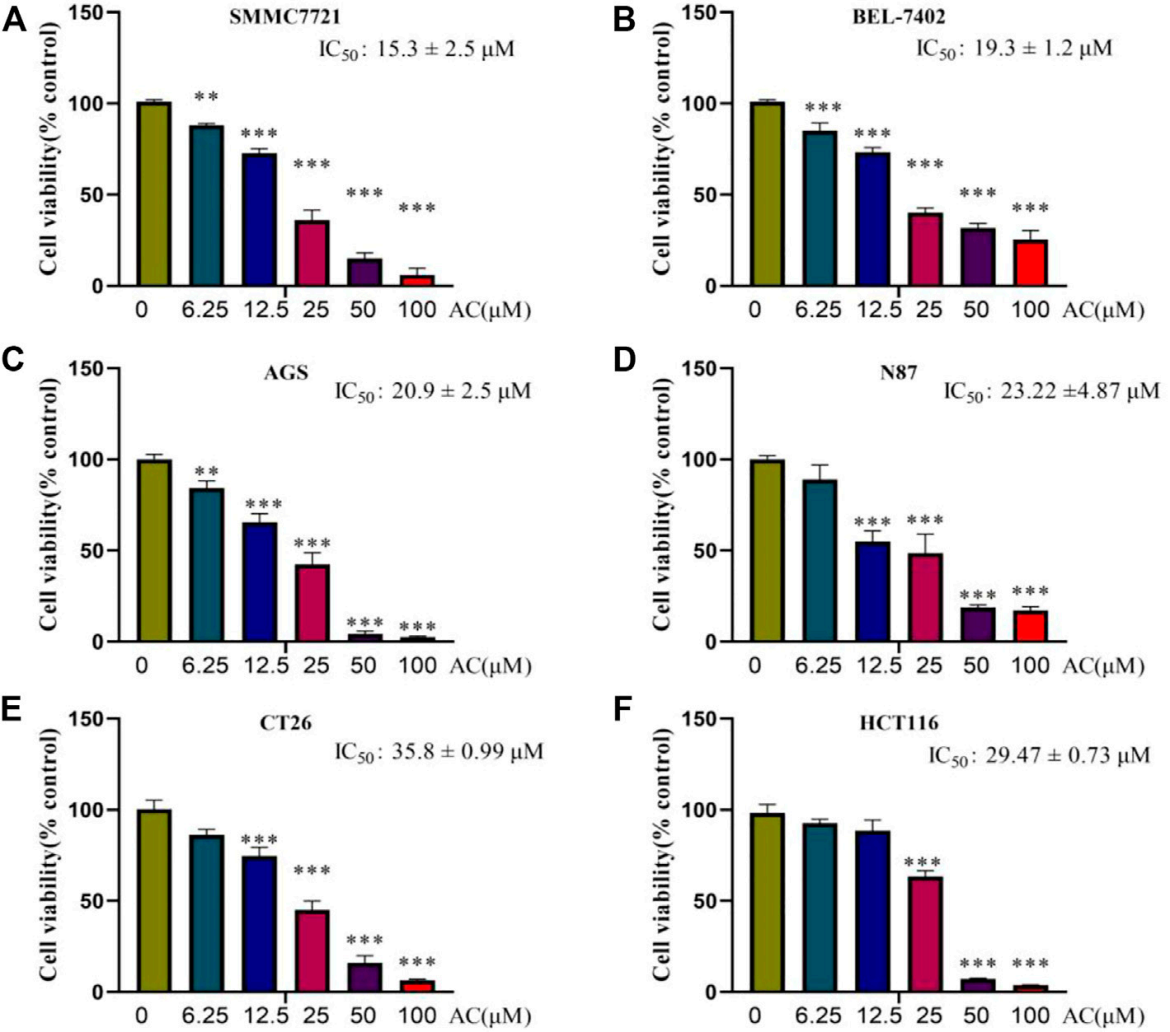
Cytotoxicity of AC on GI tumours. **(A**, **B)** The cell viability of SMMC7721 and BEL-7402 were inhibited by AC. **(C**, **D)** The cell viability of AGS and N87 were inhibited by AC. **(E**, **F)** The cell viability of CT26 and HCT116 were inhibited by AC. *p < 0.05, **p < 0.01, ***p < 0.001 (vs. 0 group).

To explore the toxicity of AC on GI tumors, liver cancer cells SMMC7721, BEL-7402, gastric cancer cells AGS, N87, intestinal cancer cells CT26, and HCT116 were selected for the study. The results of the SRB assay showed that AC could induce GI tumor death in a dose-dependent manner. AC has an inhibitory effect on the growth of SMMC7721 with IC_50_ of 15.3 ± 2.5μM, and BEL-7402 with IC_50_ of 19.3 ± 1.2 μM (Figures 2A, B). As for gastric cancer cells, the IC_50_ of AC for AGS is 20.9 ± 2.5 μM and the IC_50_ for N87 is 23.2 ± 4.87 μM (Figures 2C, D). As for intestinal cancer cells, AC’s IC_50_ for CT26 is 35.8 ± 0.99μM, and for HCT116 IC_50_ is 29.47 ± 0.73 μM (Figures 2E, F). In total, AC could inhibit the proliferation of GI tumors and exhibit smaller concentrations of IC_50_ in hepatocellular carcinoma cells.

### Flow cytometry detects the effect of AC on the behavior of hepatocellular carcinoma cells

**FIGURE 3:**
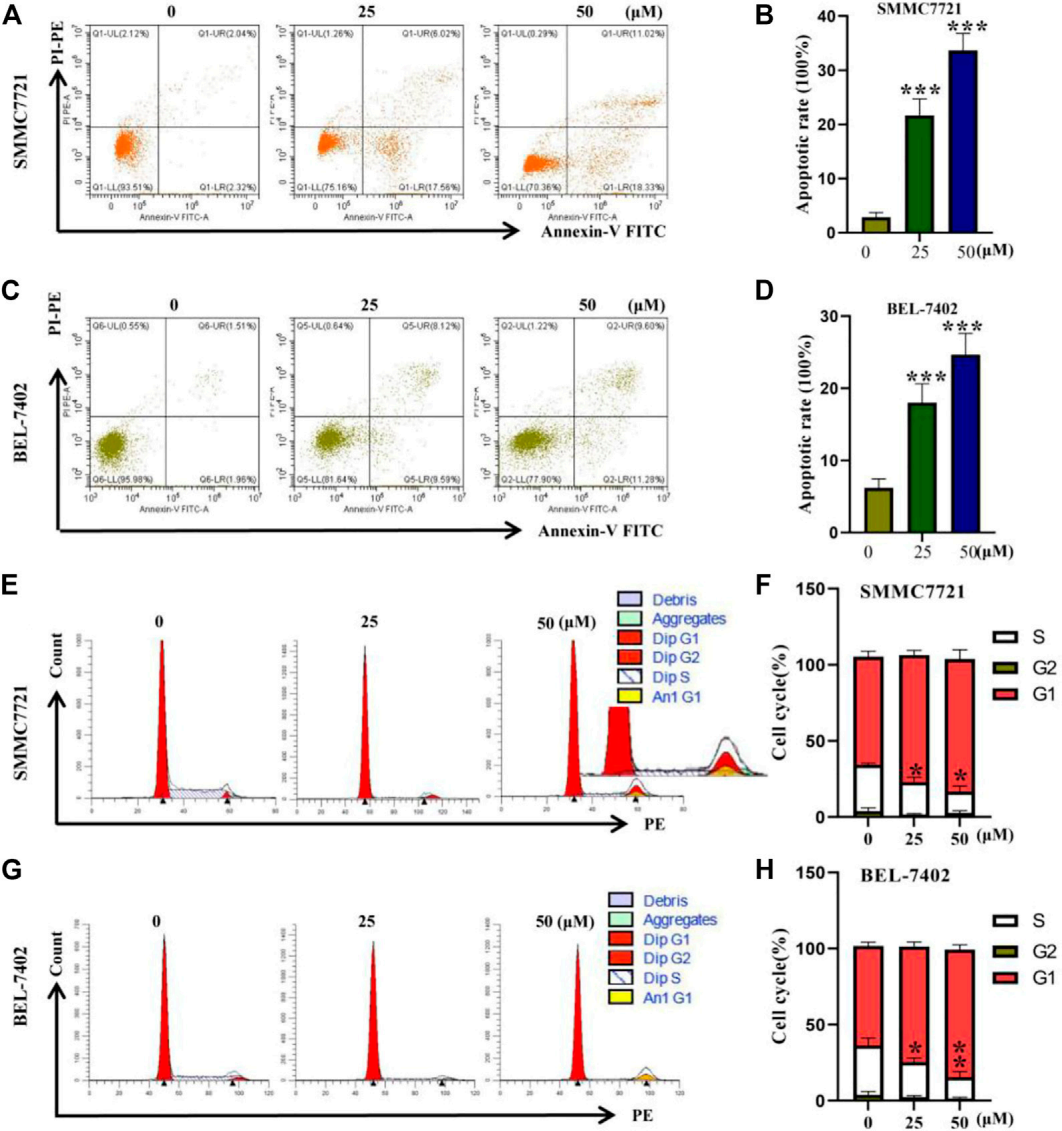
The results of flow cytometry. **(A**, **B)** Apoptosis induced by AC on SMMC7721. **(C**, **D)** Apoptosis induced by AC on BEL-7402. **(E**, **F)** Effects of AC on cell cycle in SMMC7721. **(G**, **H)** Effects of AC on cell cycle in BEL-7402. *p < 0.05, **p < 0.01, ***p < 0.001 (*vs* 0 group).

Earlier SRB cell activity assay showed that AC could inhibit SMMC7721 proliferation. And flow cytometry showed that AC could promote the death of SMMC7721 (Figures 3A, B). As shown in Figures 3C, D, AC could significantly promote BEL-7402 death with the strongest effect at 50 μM. The cell cycle of 0, 25, and 50 μM AC on SMMC7721 and BEL-7402 were detected by Flow cytometry. As for SMMC7721, 0 μM AC administered group G1 phase cells accounted for about 64% of the total cell number, and S phase cells about 30%. In the 25 μM administration group, G1 phase cells accounted for approximately 84% of the total cell number, and S phase cells approximately 10%. In the 50 μM administration group, G1 phase cells accounted for about 85% of the total cell number, and S phase cells accounted for 9% (Figures 3E, F). The effect of AC on the BEL-7402 cell cycle was similar to that of SMMC7721, with AC inducing a decrease in the BEL-7402 S phase and an increase in the G1 phase BEL-7402 (Figures 3G, H). Taken together, with the increase of the administration concentration, the number of cells in the G1 phase increased and the number of cells in the S phase decreased, indicating that the synthesis of genetic material is blocked in the G1 phase.

### Liver cancer cell death induced by AC

**FIGURE 4:**
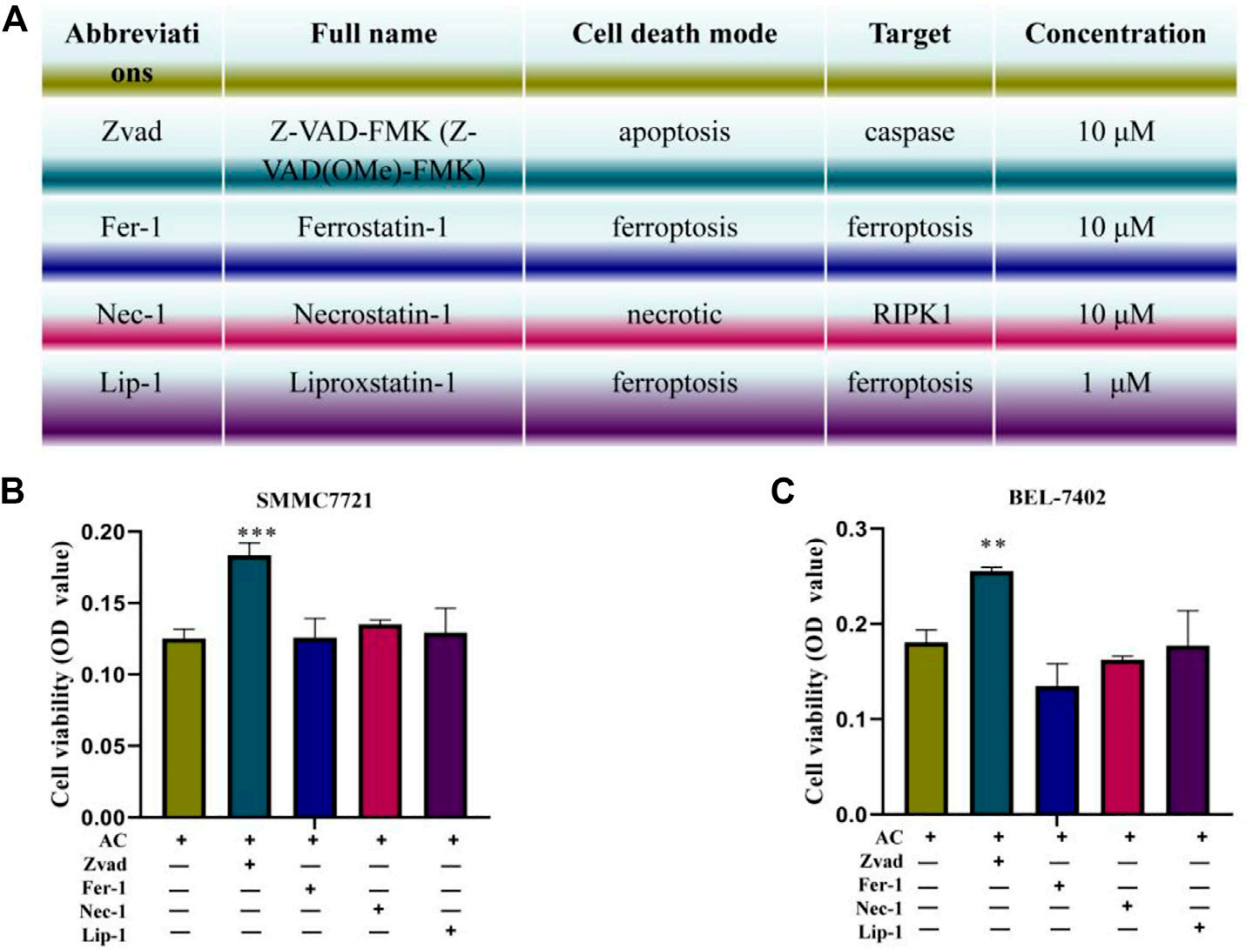
The cell death mode induced by AC. **(A)** Full names, concentrations, and targets of the inhibitors Zvad, Fer-1, Lip-1, and Nec-1. **(B**, **C)** Reverse effect of the inhibitors Zvad, Fer-1, Lip-1, Nec-1 on AC-induced death of hepatocellular carcinoma cells induced by AC. * means compared with AC group, **p < 0.01, ***p < 0.001 (*vs* AC (+), Zvad (-), Fer-1 (-), Lip-1 (-), and Nec-1 (-) group).

To further explore the cell death method of AC on hepatocellular carcinoma cells, the inhibitors Zvad, Fer-1, Lip-1, and Nec-1 were co-administered with AC. Figure 4A showed the full names, targets, and concentrations of the apoptosis inhibitor Zvad, the iron death inhibitors Fer-1 and Lip-1, and the necrosis inhibitor Nec-1. Besides, the SRB staining was used to detect the cell viability, and it was found that Zvad significantly inhibited the cell death of hepatocellular carcinoma cells caused by AC (Figures 4B, C), which advanced apoptosis of AC on hepatocellular carcinoma cells.

### AC induced hepatocellular carcinoma cell apoptosis

**FIGURE 5:**
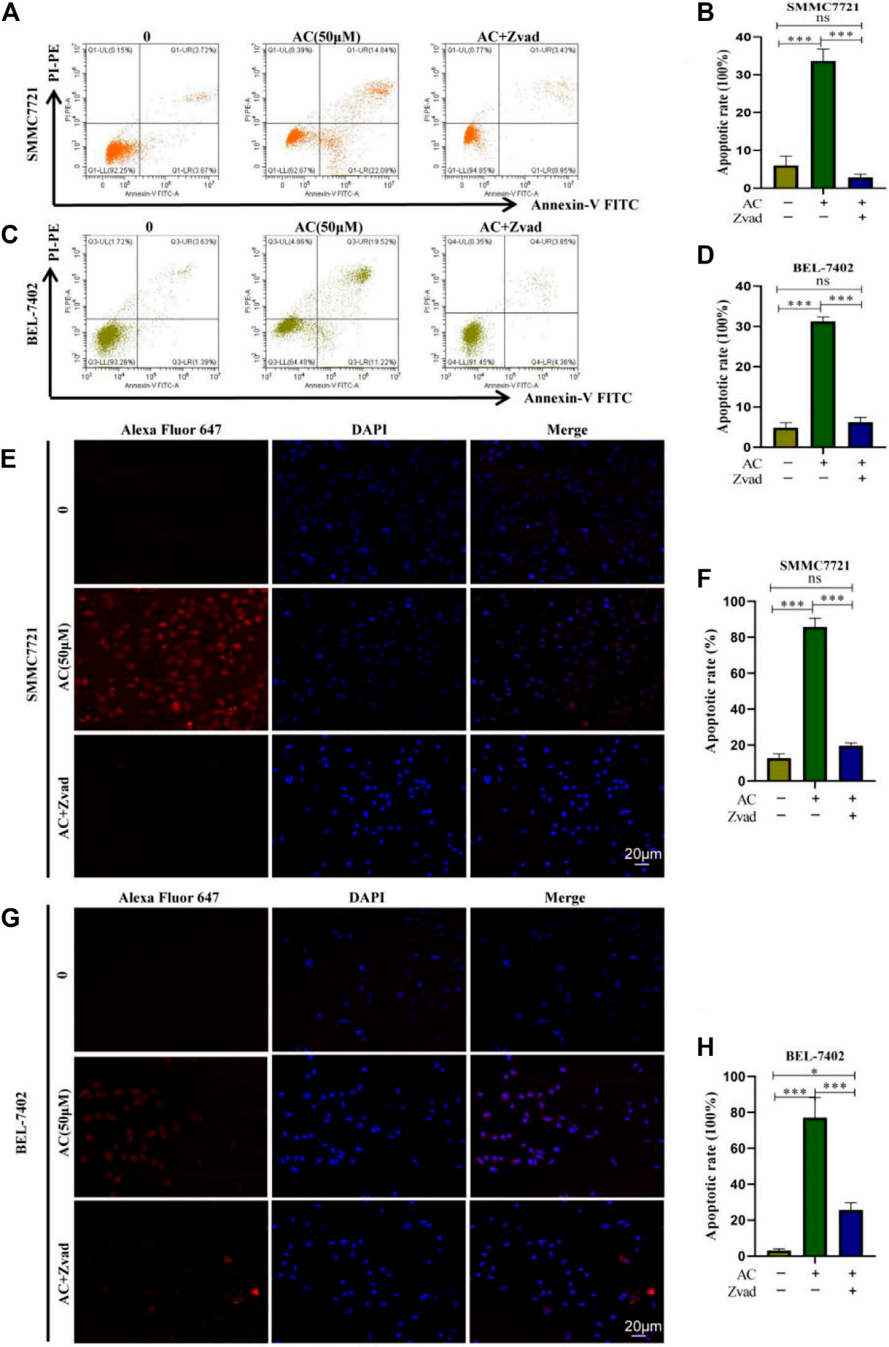
Reversal effect of Zvad on AC-induced apoptosis of hepatocytes. **(A**, **B)** Reversal effect of Zvad on AC-induced apoptosis of SMMC7721. **(C**, **D)** Reversal effect of Zvad on AC-induced apoptosis of BEL-7402. **(E**, **F)** Reversal effect of Zvad on AC-induced apoptosis of TUNEL in SMMC7721. **(G**, **H)** Reversal effect of Zvad on AC-induced apoptosis of TUNEL in BEL-7402. The image of confocal, and apoptotic cells was stained by ALEX647 (red), and the nuclei were dyed blue by DAPI. *p < 0.05; **p < 0.01; ***p < 0.001 (vs*.* 0 group).

Flow cytometry was used to detect the distribution of apoptosis cells, SMMC7721 and BEL-7402 cells were treated with AC and Zvad for 24 h. In SMMC7721, the total apoptosis rate in the AC 50 μM administration group was 23.54%, the total apoptosis rate in the Zvad + AC 50 μM group was 7.59%, and the apoptosis inhibitor was significantly reduced the total cell apoptosis rate (Figures 5A, B). For BEL-7402 cells, the total apoptosis rate in the AC 50 μM administration group was 20.28%, and the total apoptosis rate in the Zvad + AC 50 μM group was 4.01%, and apoptosis was inhibited (Figures 5C, D). AC significantly reduced the total cell apoptosis rate. In addition, the TUNEL assay results showed that the red fluorescence of the AC 50 μM with Zvad 10 μM combined administration group was significantly weaker than the 50 μM administration group in SMMC7721(Figures 5E, F). As shown in Figures 5G, H, similar to the results of SMMC7721, the red fluorescence was stronger in the AC group compared with the AC 0 group. Compared with the AC group, the red fluorescence diminished when AC was combined with Zvad. Therefore, it is proved that AC can induce hepatocellular carcinoma cells to apoptosis, and this reaction can be reversed by the apoptosis inhibitor Zvad.

### The influence of AC on apoptosis-related proteins

**FIGURE 6:**
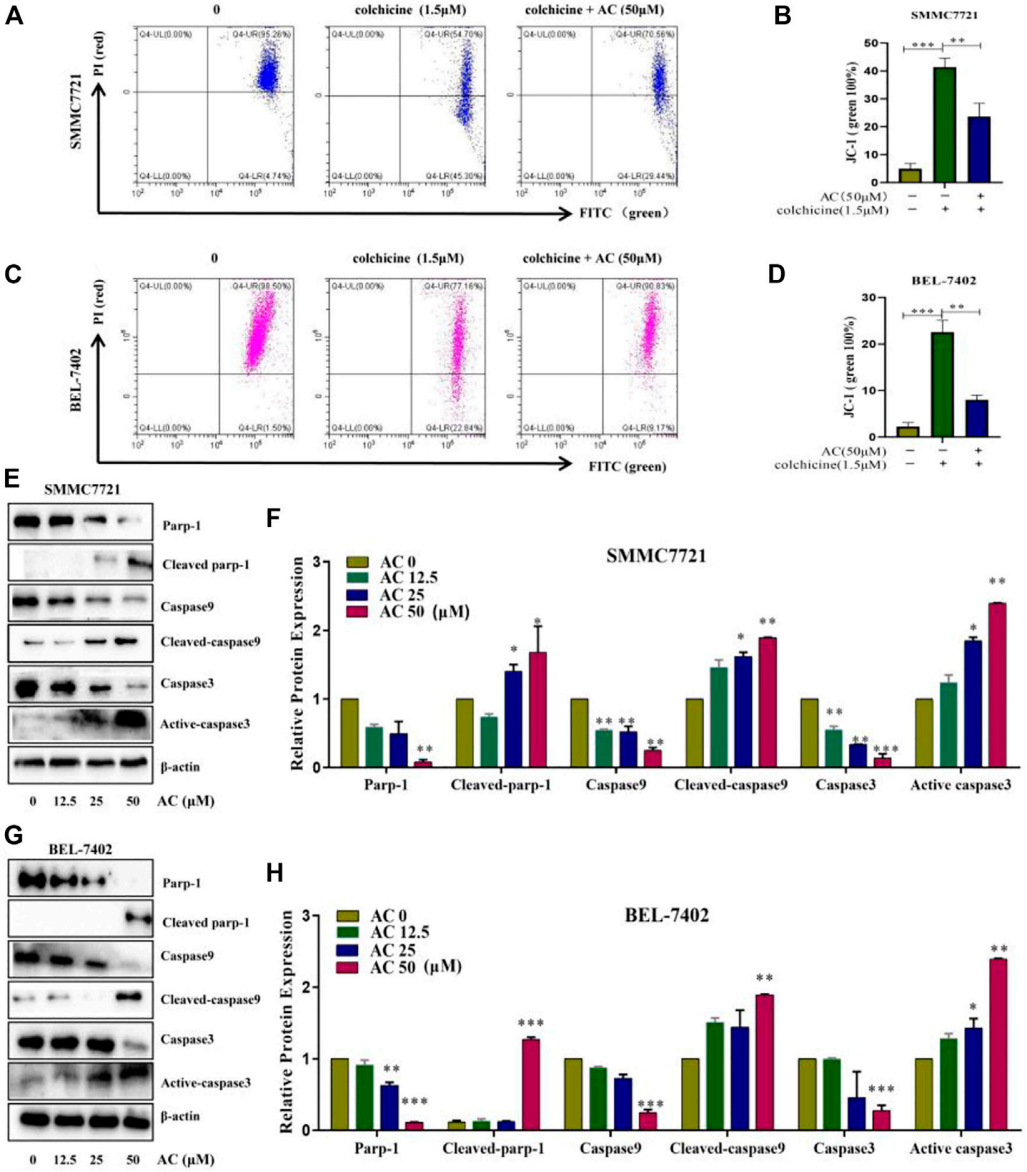
Effects of AC on the apoptosis-related signaling pathway proteins. **(A**, **B)** Effect of AC on mitochondrial membrane potential in SMMC7721. **(C**, **D)** Effect of AC on mitochondrial membrane potential in BEL-7402. **(E**, **F)** Western blot detection of Parp-1, Cleaved-parp-1, Caspase9, Cleaved-caspase9, Caspase3, and Active-caspase3 expression and statistical results in SMMC7721. **(G**, **H)** Western blot detection of Parp-1, Cleaved-parp-1, Caspase9, Cleaved-caspase9, Caspase3, and Active-caspase3 expression and statistical results in BEL-7402. *p < 0.05; **p < 0.01; ***p < 0.001 (vs*.* 0 group).

**FIGURE 7:**
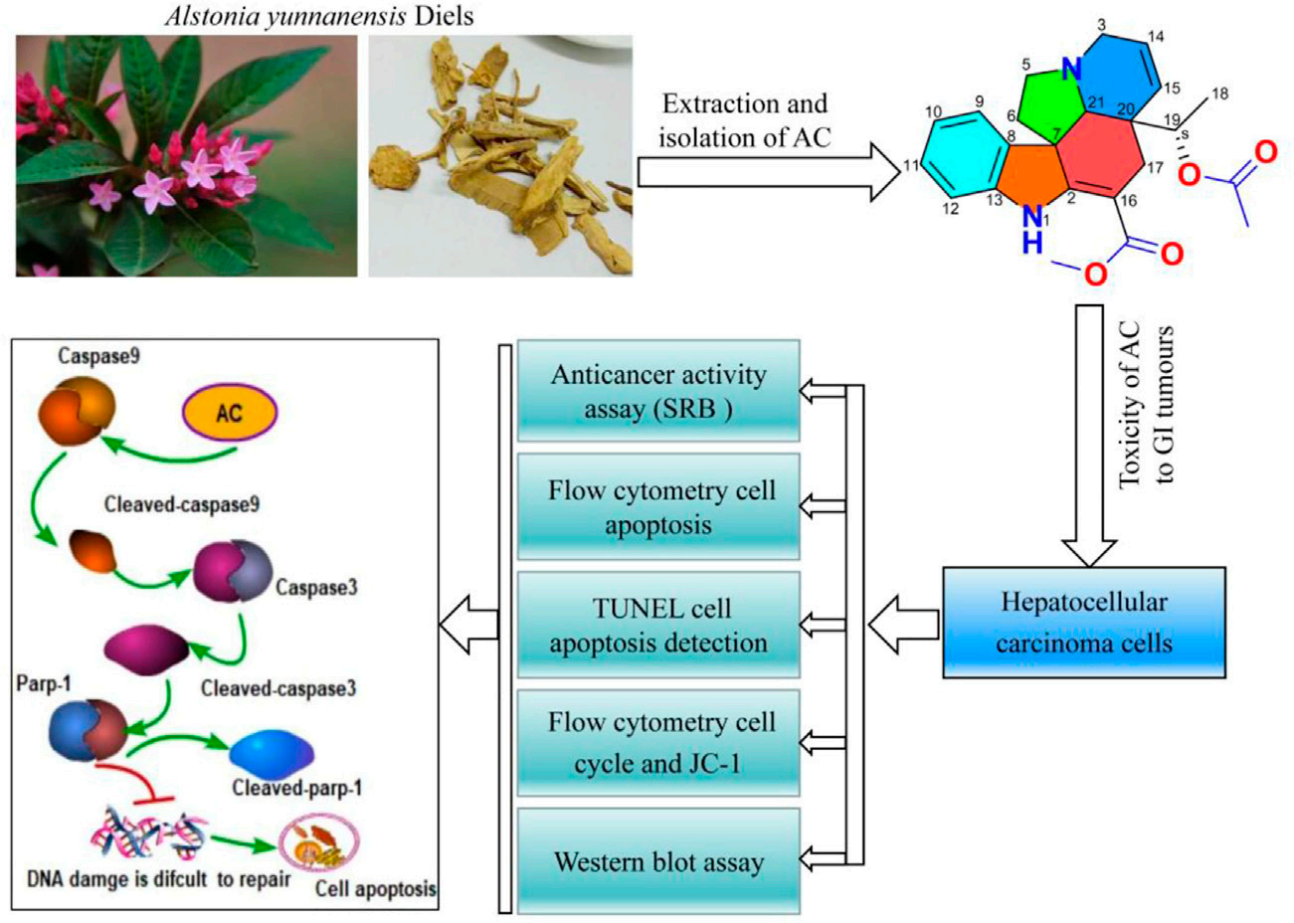
Graphical abstract of this study. Isolation, identification and biological activity of AC, and the effect of AC on apoptosis-related proteins.

The flow cytometry results showed that the mitochondrial membrane potential, which was reduced due to the action of colchicine, was increased under the action of AC, indicating that AC can effectively increase the mitochondrial membrane potential and enhance the exchange of substances inside and outside the mitochondria (Figures 6A–D). Mitochondria are closely associated with apoptosis, which also suggests that AC can influence mitochondrial apoptotic mechanisms. Earlier experiments had shown that apoptosis inhibitor Zvad significantly inhibits AC, so we then examined the caspase-related pathway (Zvad targets) using a western blot. Hepatocellular carcinoma cells were treated with different concentrations of AC for 24 h, and the expression Parp-1, Cleaved-parp-1, Caspase-9, Cleaved-caspase9, Caspase3, and Active-caspase3 were analyzed. As for SMMC7721, we found that Parp-1, Caspase9, and Caspase-3 decreased with the increase of AC, while Cleaved-parp-1, Cleaved-caspase9, and Active-caspase3 increased (Figures 6E, F). AC on caspase pathway proteins in BEL-7402 showed similar results to SMMC7721, with caspase9, caspase3, and Parp-1 all cleaved and activated to subunits (Figures 6G, H). A general schematic of the isolation and activity testing of AC was shown in Figure 7. These experiments show that AC can cause the cascade activation of Caspase9-Caspase3, which further leads to the cleavage of parp-1 to prevent DNA damage repairment and lead to cell apoptosis.

## Disscussion

Liver cancer is a common GI malignant tumor with high morbidity and mortality, which seriously threatens human health and life ^(6, 7)^. According to previous studies, the incidence of liver cancer has declined to varying degrees in both the United States and China, but liver cancer remains the top-ranked cause of cancer death (Xia, Dong et al., 2022). Although sorafenib and regorafenib can improve the overall survival rate of liver cancer patients, they are easy to develop drug resistance, and the prognosis is still poor (Man, Luo et al., 2021). Increasing studies have proved that NPs kill tumor cells through a variety of molecular mechanisms, including cell DNA damage and apoptosis, such as matrine (Li, Lai et al., 2016) and curcumin (Zhao, Chen et al., 2015). In addition, Dai (Fu, Xie et al., 2022) reported anti-colorectal cancer effects of seaweed-derived bioactive compounds; Kang (Lee, Jayawardena et al., 2022) reported that marine algae extracts could alleviate high-fat-induced obesity. In the present study, we isolated 32.5 mg of the monomeric compound AC from the *Alstonia yunnanensis* Diels roots. The absolute configuration of AC was identified as 19(s), 20(s) -Acetoxytabernosine as an indole alkaloid by spectroscopy. Pharmacological activity assays showed that AC inhibited the proliferation of GI tumors, and the IC50 of AC against SMMC7721 and BEL-7402 were 15.3 ± 2.5 μM and 19.3 ± 1.2 μM. Moreover, AC could block DNA synthesis in hepatocellular carcinoma cells, with a significant decrease in S-phase and an increase in G1-phase. Mechanistic studies showed that AC could induce apoptosis in a caspase-dependent manner in hepatocytes.

*Alstonia yunnanensis* Diels is a kind of folk medicine in the Yunnan area, worth exploring the biological activity of active compounds^(37, 38)^. The chemical composition of *Alstonia yunnanensis* Diels has been studied by a larger number of scholars and is rich in indole alkaloids, and numerous studies have reported the anti-inflammatory and anti-tumor activity of these alkaloids, offering great potential in the field of anti-tumor (Cao, Liang et al., 2012). AC is the active compound of *Alstonia yunnanensis* Diels, and we have reported for the first time *in vitro* that AC inhibits the proliferation of hepatocellular carcinoma cells with a dose-dependent relationship. In addition, the cell cycle regulates the process of cell proliferation, replication, and division (Cloonan, Brown et al., 2008). The G1 phase synthesizes proteins related to DNA synthesis, the S phase is DNA synthesis, and the G2 phase synthesizes some proteins in preparation for the M phase (Cloonan, Brown et al., 2008). The cell cycle assay showed that AC induced a decrease in the S phase and an increase in the G1 phase. Taken together, these results suggest that AC-induced G1 phase blocks in hepatocellular carcinoma cells.

The SRB cell viability assay identified 4 death inhibitors against the cytotoxicity of AC and found that AC induced hepatocellular carcinoma cell death through apoptosis. The flow cytometry and TUNEL assays were also consistent with the SRB assay, further verifying that apoptosis is the core mode of AC-induced hepatocellular carcinoma cell death, and thus apoptosis-related pathways should be given more attention. Apoptosis is one of the common forms of cell death and usually results in DNA damage, blocked normal replication of DNA, and slow cell proliferation (Cohen and Rothmann, 2001). Apoptosis is a physiological form of cell death, usually divided into internal and external pathways (Sartorius, Schmitz et al., 2001). The extrinsic pathway is caused by the tumor necrosis factor receptor (TNF-α) on the cell surface and the death receptor Fas(Waring and Müllbacher, 1999; Sartorius, Schmitz et al., 2001). The other is the intrinsic pathway or the mitochondrial pathway (Sartorius, Schmitz et al., 2001). The intrinsic pathway is usually caused by DNA damage from UV exposure, and cytotoxic drugs and is characterized by disruption of mitochondrial membrane integrity and is regulated by Bcl-2 protein family members (Kirkin, Joos et al., 2004; Sharpe, Arnoult et al., 2004).

The intrinsic apoptosis pathway can usually be divided into caspase-dependent or caspase-independent (Joubert, Werneke et al., 2012). Caspase-9 is the initiator of apoptosis and acts as a focal point for multiple protein kinase signaling pathways that regulate apoptosis (Li, Nijhawan et al., 1997). Caspase-3 is the main actor of apoptosis, various apoptosis-stimulating factors initiate the cleavage of Caspase-3 by different proteases, leading to its activation and further cleavage of different substrates, leading to the amplification of the protease cascade and cell death (Liu and Linn, 2000). PARP is a cleavage substrate for caspase, a core member of apoptosis, and plays an important role in DNA damage repair and apoptosis (Nicholson, Ali et al., 1995). Caspase-9 and Caspase-3 are usually activated in the apoptosis pathway [10-11], which causes the cleavage of the PARP protein of tumor cells [12-13], thereby inhibiting the growth of cancer cells (Farha, Dhanya et al., 2014). Khan (Khan, Siveen et al., 2018) reported that curcumin could promote apoptosis in human head and neck cancer cells by dose-dependently activating cleavage of Caspase 3, Caspase 9, and PARP. These experimental results prove that AC can significantly activate Caspase-9, thereby further activating the key enzyme Caspase-3 for cell apoptosis. Caspase-3 plays a decisive role in caspase-dependent cell apoptosis. The sequential activation of Caspase-3 can lead to the cleavage of Parp-1, causing Parp-1 to lose enzyme repair activity, accelerating cell instability, and making it difficult to repair DNA, ultimately leading to cell apoptosis.

The natural product AC demonstrated better killing of hepatocellular carcinoma cells *in vitro*, further validating the anti-tumor activity of indole alkaloids. NPs serve as a major treasure trove of drug sources, these chemical structures are complex and thus NPs can be an important source of lead compounds and drug candidates (Amin, Kucuk et al., 2009). However, there are still shortcomings in the development of drugs from NPs. The extracted NPs are often low in content, the complexity of the structure makes them difficult to synthesize, and some NPs have therapeutic activity but are highly toxic. Of course, modifying and optimising the NPs to obtain a suitable drug lead or drug candidate would be an effective strategy to tap natural product resources for the development of anti-tumor drugs (Ross and Hoye, 2017). Additionally, synthetic biology (Guo, Wang et al., 2022), nano-drug delivery (Kim, Moore et al., 2015), and combination drug delivery (Mekkawy, Eleraky et al., 2022) can solve the difficulties of synthesizing NPs, improve their bioavailability and reduce their toxic effects. These new scientific and technological developments will continue to lead to a greater role for NPs in the development of anti-tumor drugs.

## Conclusion

In summary, this study determined for the first time that AC induces apoptosis of liver cancer cells SMMC7721 and BEL-7402 through the caspase-dependent pathway, and found that the synthesis of genetic material is blocked in the G1 phase, resulting in a decrease in DNA replication in the S phase (Figure 7). The monomer compound AC has enriched the research on the chemical composition of *Alstonia yunnanensis* Diels. At the same time, the application of NPs to medical research can help the development of traditional national medicines, and it is helpful for human health and disease treatment. However, the amount of monomer compounds isolated in this study is relatively limited, so *in vivo* experiments have not been carried out. Next, the monomer compound AC will be enriched, its specific mechanism of apoptosis will be discussed in-depth, and animal experiments will be supplemented.

## Data Availability

The original contributions presented in the study are included in the article/Supplementary Material, further inquiries can be directed to the corresponding authors.
