# Supplementary material for: Alkaloid from Alstonia yunnanensis diels root against gastrointestinal cancer: Acetoxytabernosine inhibits apoptosis in hepatocellular carcinoma cells
Source: Front Pharmacol. 2023 Jan 11;13:1085309. doi: 10.3389/fphar.2022.1085309 (PMC9873973; doi:10.3389/fphar.2022.1085309)
Supplement: Supplementary file 1 [file DataSheet1.docx]

**Alkaloid from *Alstonia yunnanensis* Diels root against Gastrointestinal Cancer: Acetoxytabernosine inhibits apoptosis in hepatocellular carcinoma cells**

Qi Lai^1&^, Chun-Ju Yang^1&^, Qi zhang^1^, Min Zhuang^1,2^, Yan-hua Ma^1,2^, Cheng-Yuan Lin*^2,3^, Guang-Zhi Zeng*^1^, Jun-Lin Yin*^1^

1. Key Laboratory of Chemistry in Ethnic Medicinal Resources, State Ethnic Affairs Commission & Ministry of Education; School of Ethnic Medicine, Yunnan Minzu University, Kunming 650500, China.
2. Centre for Chinese Herbal Medicine Drug Development Limited, Hong Kong Baptist University, Hong Kong SAR, China
3. School of Chinese Medicine, Hong Kong Baptist University, Hong Kong SAR, China

&Co-first authors:

Qi Lai and Chun-Ju Yang.

*Corresponding author:

Jun-Lin Yin, PHD, E-mail: yinjunlin1979@sina.com.

Guang-Zhi Zeng, PHD, E-mail: g.zh_zeng@163.com.

Cheng-Yuan Lin, PHD, E-mail: lincy@hkbu.edu.hk.

Supporting information available: 1D and 2D NMR spectra were recorded on a Bruker AVANCE 400 NMR spectrometer. The spectrum data of AC are shown in and Figures S1-S8.

Figure S1. ^1^H NMR spectrum of AC in CDCl3

Figure S2. ^13^C NMR spectrum of AC in CDCl3

FigureS3. HSQC spectrum of AC in CDCl3

Figure S4. HMBC spectrum of AC in CDCl3

Figure S5. COSY spectrum of ac in CDCl3

Figure S6. ^1^H-^1^HCOSY and HMBC

Figure S7. The mass spectrometry of AC ([M+H]^+^, [M+Na]^+^)

Figure S8. UV-visible spectroscopy of AC

NMR data (BrukerAV, Germ): ^1^H-NMR (400 MHz, CDCl3) δ 8.99 (s, 1H, N-H), 7.27 (s, ^1^H, H-9), 7.21 (t, J = 6.4 Hz, 1H, H-11), 7.16 (td, J = 7.7, 1.2 Hz, 1H, H-12), 6.94 – 6.79 (m, 1H, H-10), 5.94 (ddd, J = 10.0, 4.9, 1.5 Hz, 1H, H-14), 5.82 – 5.73 (m, 1H, H-15), 3.54 – 3.42 (m, 1H, H-3a), 3.28 – 3.19 (m, 1H, H-3b), 3.09 – 3.01 (m, 1H, H-5b), 2.80 – 2.76 (m, 1H, H-5a), 2.52 – 2.40 (m, 1H, H-7), 1.83(1H, m, H-6), 1.81 (s, 3H, CH3CO2), 0.92 (d, J = 6.4 Hz, 3H, Me-18). ^13^C-NMR (100 MHz, CDCl3) δ 169.98 (CH3CO2), 168.45 (CO2CH3), 166.05 (C-2), 143.36 (C-13), 137.52 (C-8), 129.66 (C-15), 127.92 (C-11), 126.87 (C-14), 121.26 (C-9), 120.74 (C-10), 109.76 (C-12), 91.72 (C-16), 69.62 (C-19), 66.60 (C-21), 55.46 (C-7), 50.96 (CO2CH3), 50.91 (C-5), 50.16 (C-3), 45.73 (C-20), 44.33 (C-6), 27.39 (C-17), 20.79 (CH3 CO2), 15.34 (C-18).


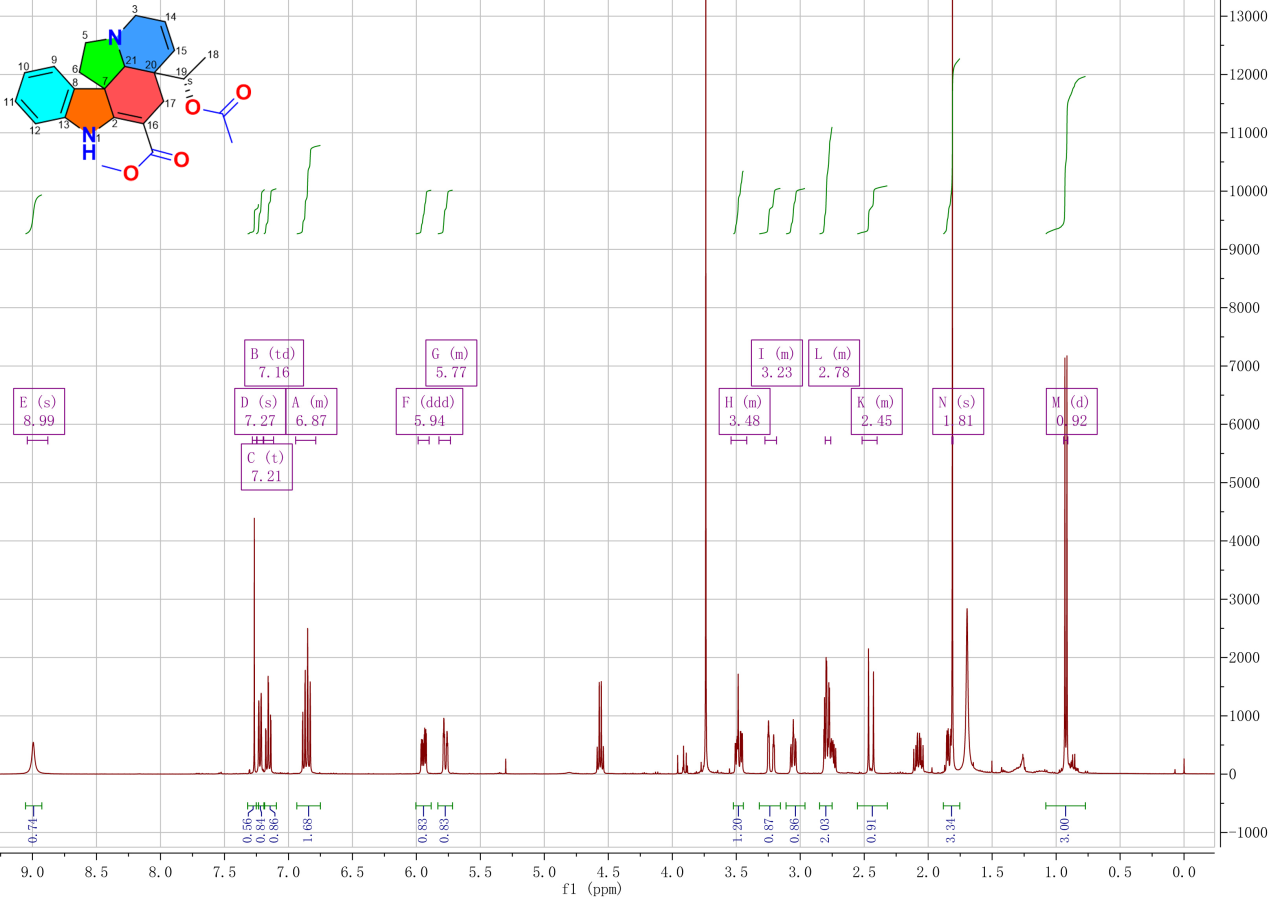


Figure S1. ^1^H NMR spectrum of AC in CDCl3


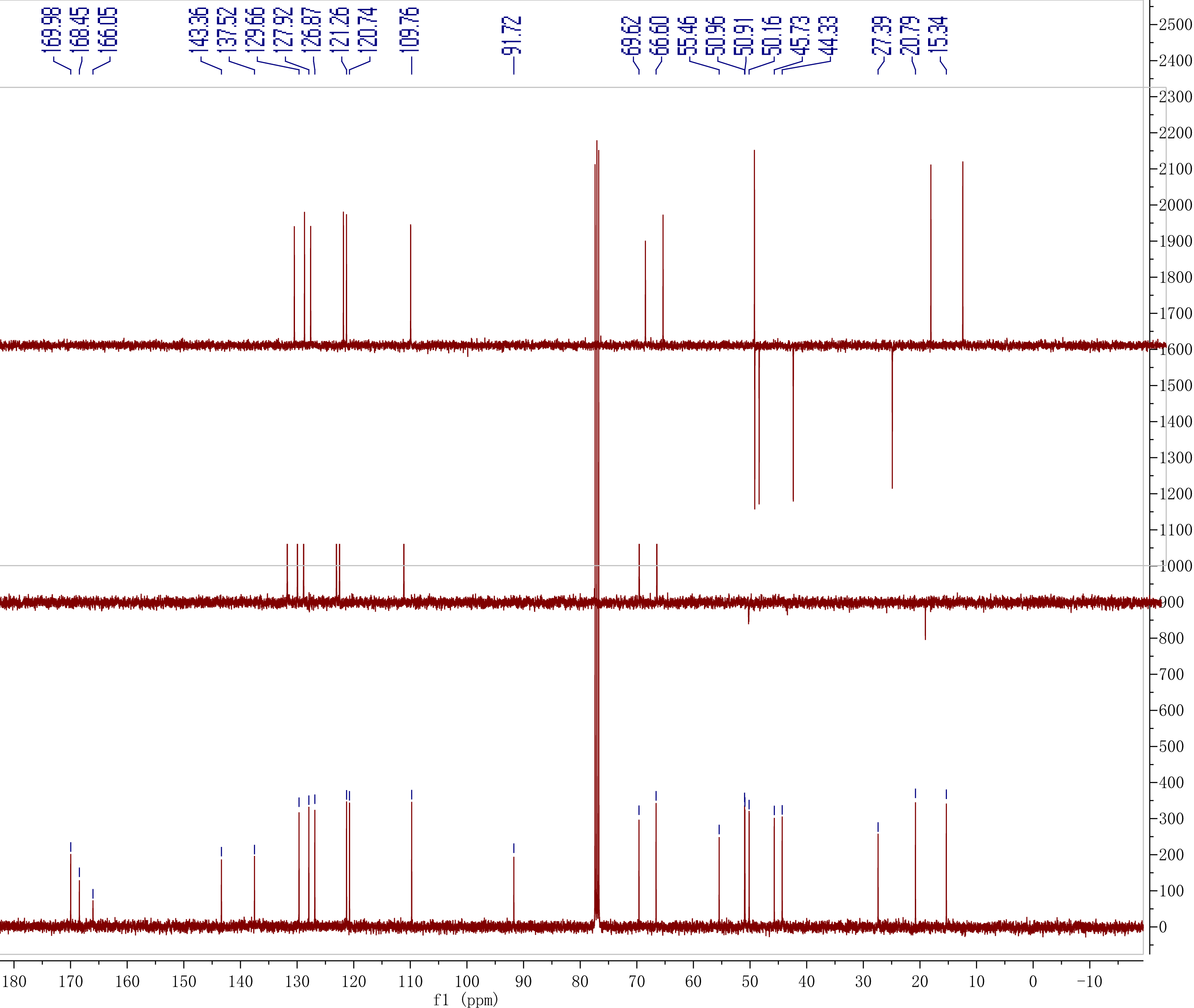


Figure S2. ^13^C NMR spectrum of AC in CDCl3


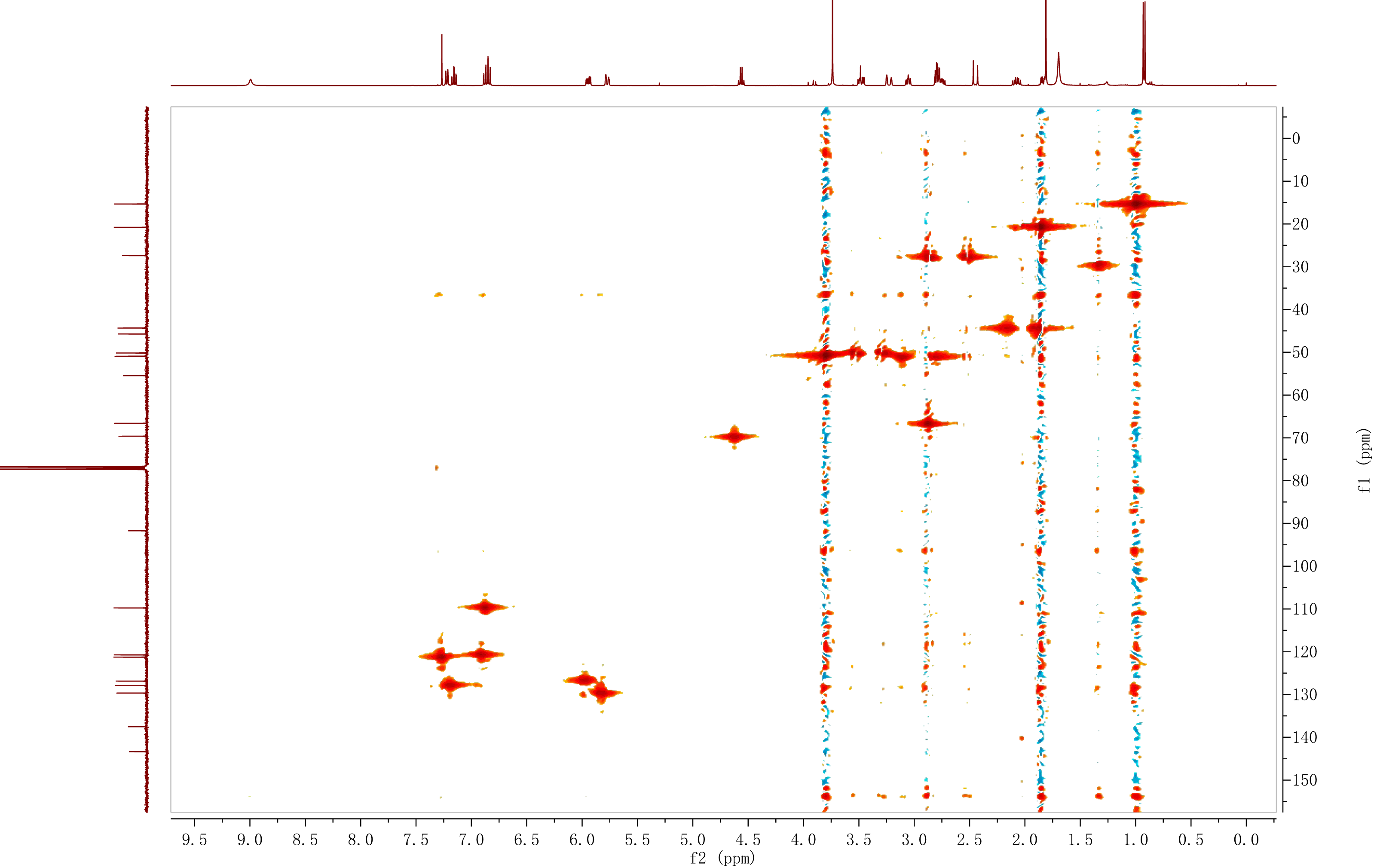


FigureS3. HSQC spectrum of AC in CDCl3


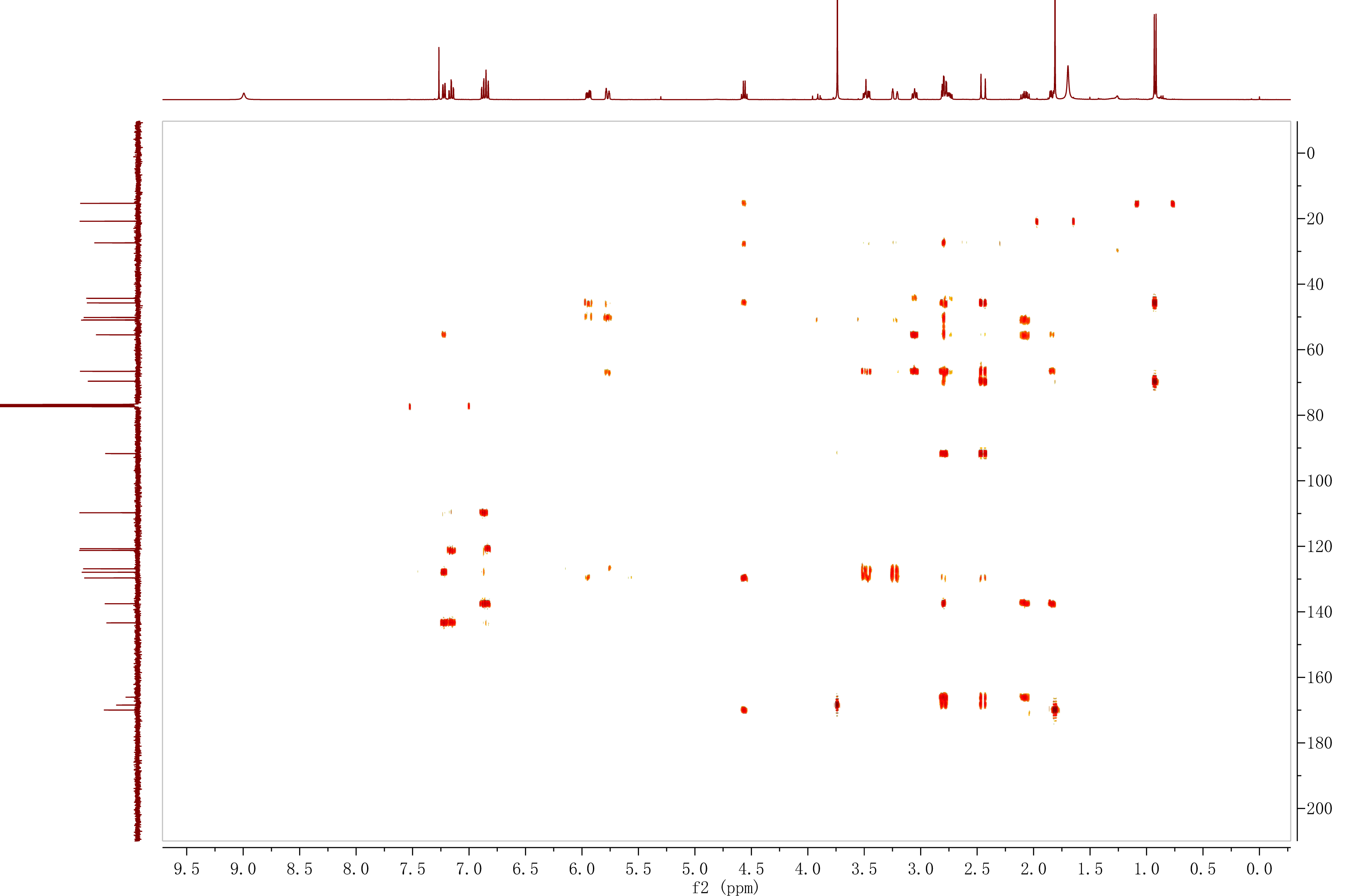


Figure S4. HMBC spectrum of AC in CDCl3


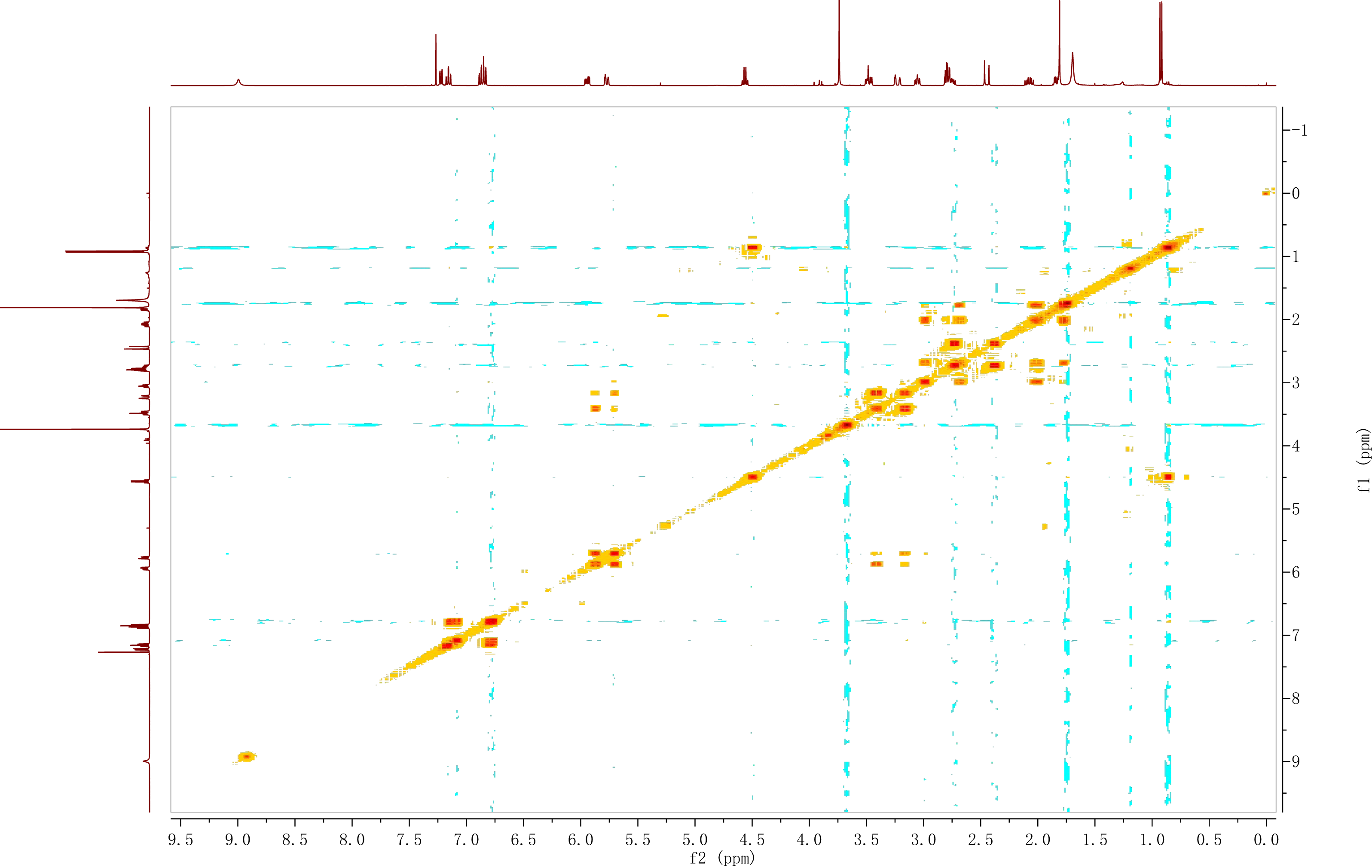


Figure S5. COSY spectrum of AC in CDCl3


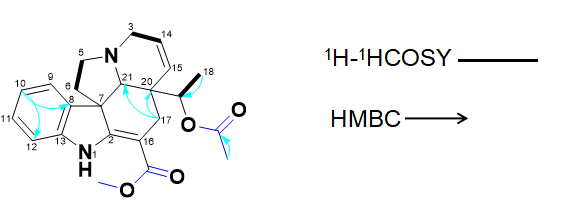


Figure S6. ^1^H-^1^HCOSY and HMBC correlations of compound AC


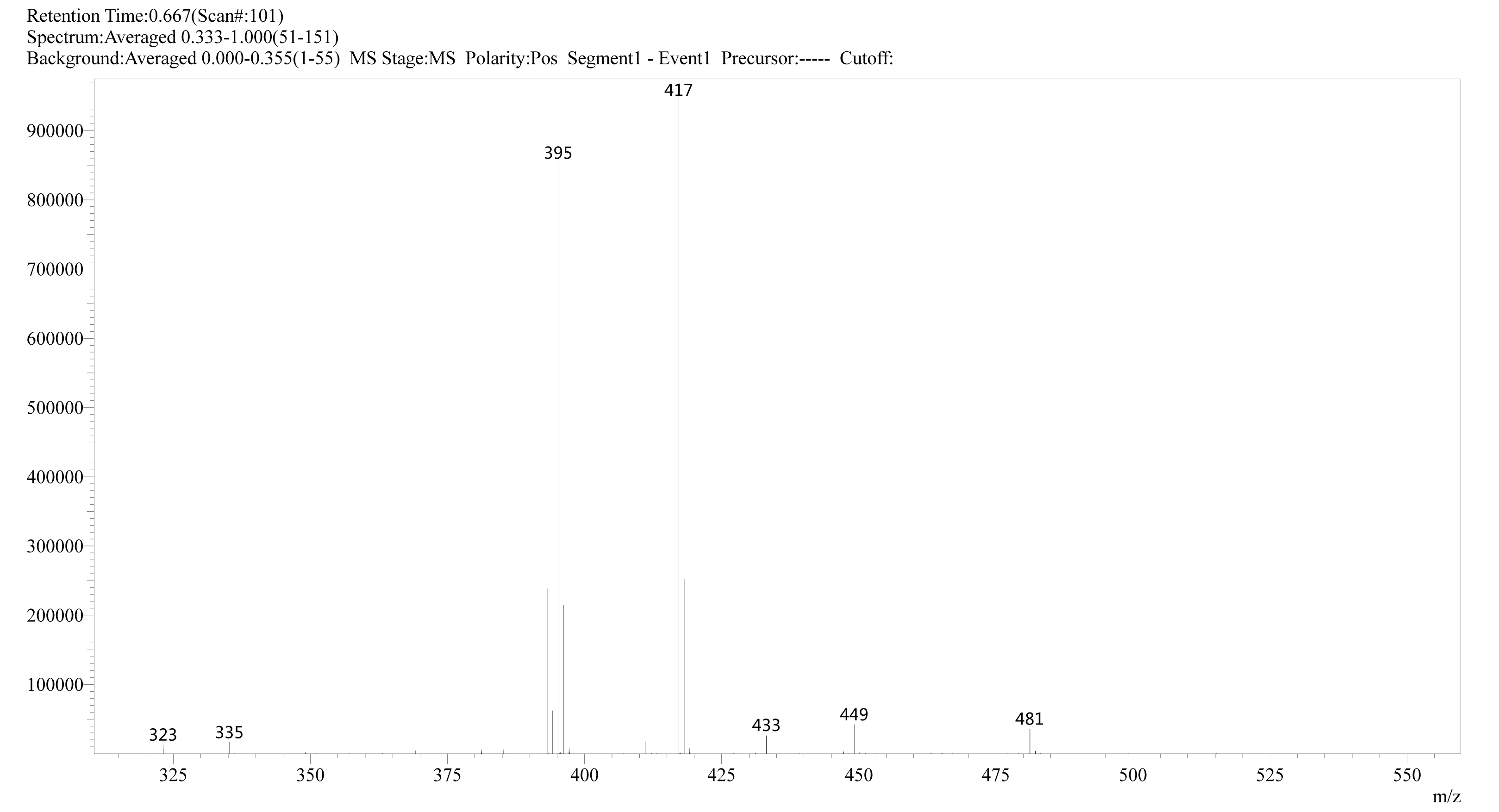


Figure S7. The mass spectrometry of AC ([M+H]+, [M+Na]+)





Figure S8. UV-visible spectroscopy of AC
